# Diagnostic value of multislice spiral computed tomography (CT) combined with CT angiography for intra-abdominal undescended testis secondary seminomas

**DOI:** 10.1186/s40644-019-0210-z

**Published:** 2019-05-16

**Authors:** Si-qi Wang, Fang-yuan Ren, Jian-hua Wang, Zhi-hao Ren, Zhong-gao Jin, Yu Xu, Zhen-hui Li, Qian-jiang Ding, Chao Zhong

**Affiliations:** 1 Department of Radiology, The Affiliated Hospital of Medical school, Ningbo University, Zhejiang, 315020 China; 2 Medicine School of Ningbo University, Zhejiang, 315020 China; 3 Ningbo No.7 hospital, Zhejiang, 315010 China; 4 Yunnan Cancer Hospital, Kunming, 650118 Yunnan Province China

**Keywords:** CTA, CT; seminoma, Intra-abdominal undescended testis

## Abstract

**Objective:**

To discuss the diagnostic value of multislice spiral tomography (CT) combined with CT angiography (CTA) technology in intra-abdominal undescended testis secondary seminoma cases.

**Methods:**

We retrospectively analyzed the CT and CTA imaging features of CT and CTA findings of nine patients with an intra-abdominal undescended testis secondary seminoma.

**Results:**

The tumors in all nine patients were mainly solid, and the average CT value was 38.4 ± 3.4 HU. Low-density areas of various sizes were visible in the tumors, and calcifications were detected in two patients. The tumors in eight patients had a complete capsule, which pressed on the surrounding structures. In one patient, the tumor had an incomplete capsule, which invaded the surrounding structures. Some of the solid tumors showed progressive and slight enhancement on the CT-enhanced scans. The values in the arterial phase, venous phase, and delayed phase were 46.3 ± 5.1 (40–55 HU), 57.3 ± 7.3HU (48–68 HU), and 65.1 ± 7.2HU (56–77 HU), respectively, with an average increase rate of 27.0 ± 7.2 HU. No enhancement was found in low-density areas on the CTA scans, and the supply arteries of the tumors in the nine patients all originated from the abdominal aortic wall 2–3 cm below the renal ostia. These arteries became thickened and tortuous when near the tumors, and there were no branching vessels. In eight patients, the supply arteries of the tumors originated from the posterior tumor and ended inside the tumor, and they originated from anterior of the tumor in one patient. Testicular venous drainage was detected in three patients, and lymph node metastasis in the abdominal aorta detected in two cases.

**Conclusion:**

An intra-abdominal undescended testis secondary seminoma exhibits a characteristic appearance on CT. CTA shows a three-dimensional testicular vascular pedicle sign of a seminoma. A combination of CT and CTA can improve the diagnostic accuracy of an intra-abdominal undescended testis secondary seminoma.

## Introduction

An undescended testis, which is also called cryptorchidism, can be divided into intraperitoneal and inguinal types [1]. The incident rate of malignant tumors of undescended testes is 20–46 times higher than malignant tumors of scrotal testes, and the incident rate of malignant tumors of intraperitoneal undescended testes is six times higher than malignant tumors of groin or scrotal testes [2]. Ueno [3] reported that seminomas accounted for 60% of undescended testicular malignant tumors, whereas pure seminomas accounted for 90% of cases. An intra-abdominal giant seminoma is rare and is mainly caused by an intraperitoneal undescended testis malignancy [4]. A familial history of giant seminomas can aid the diagnosis of an undescended testis [5] . However, not all patients can provide a medical history. Furthermore, intra-abdominal giant seminomas may be present in a variety of retroperitoneal diseases [6, 7]. In addition, due to the large volume of a giant seminoma, it is difficult to diagnose the characteristics and origin of the tumor. Hence, an accurate diagnosis of the imaging features of intra-abdominal giant seminomas is very important for treatment and prognosis [8]. Although previous studies have described intra-abdominal undescended testis secondary seminoma cases, information on their imaging features are lacking [9, 10].

From January 2010 to December 2017, we analyzed multislice spiral tomography (CT) combined with CT angiography (CTA) data on nine cases of intra-abdominal giant seminomas confirmed by surgery and pathology. Volume rendering (VR) and curved planar reformation (CPR) were used to clearly display the supply arteries and the origin, number, and distribution of the arteries. These results were compared with postoperative pathological results to assess the diagnostic value of computed tomography (CT) combined with CT angiography (CTA) for intra-abdominal undescended testis secondary seminomas.

## Material and methods

### Clinical information

From January 2010 to December 2017, data on nine patients with intra-abdominal undescended testis secondary seminomas confirmed by surgery and pathology were collected from the Affiliated Hospital of Ningbo University, Ningbo No. 7 hospital and Yunnan Province Tumor Hospital. All patients signed an inform consent form. All the patients were males, aged between 24 and 54 y, with an average age of 39.6 y. The patients were mainly from rural China and from a poor socioeconomic background, and the pathology had not been detected in childhood. An intra-abdominal undescended testis secondary seminoma was diagnosed based on abdominal discomfort or a palpable abdominal mass or other abdominal disease on an imaging examination.

### CT examination

The examination was performed using a 256-slice CT scanner (Brillance iCT, Philips Healthcare, Eindhoven, The Netherlands) and a 16-row CT scanner (Brillance, Philips Healthcare). A CT scan was performed in all patients after they had fasted for 4 h. Each patient consumed 500–1000 ml of water orally 30 min before the examination to fill the intestinal space.

A venous indwelling needle (20 G) was embedded in the elbow vein, and the CT scan was performed with the patient in a supine position. The scan parameters were as follows: 120 KV, 180–250 mAs, thickness of 5 mm, interlamellar spacing of 5 mm, and reconstruction thickness of 1 mm. A nonionic contrast agent (100 ml) was injected intravenously. Then, 0.9% sodium chloride solution (30 ml) was injected intravenously. An automatic trigger was used, and the region of interest was placed at the level of the abdominal aorta. After the injection of the contrast agent, scanning was started automatically when the intra-arterial threshold reached 120 Hu.

### Postprocessing of the images

The reconstructed data were transferred to an EBW4.5 workstation for post-treatment. A range of reconstruction methods were used to observe the starting site and variations in the direction of the supply artery. The reconstruction methods included VR, maximum intensity projection (MIP), multiplanar reformation (MPR), CPR, and SSD. To clearly display the anatomy of the tumor and the supply artery, large vessels were removed using auto software or artificial shear. The main diagnostic criteria of a supply artery were 1) arterial thickening and enlargement; a reticular or dendritic distribution, and 2) distribution around the tumor edge consistent with tumor morphology. In cases of multiple supply arteries, the thickest artery that originated from an adjacent large vessel was identified as the supply artery.

### Pathological analysis

In seven of the nine patients, the tumors were resected, followed by general observations, microscopic observations, and immunohistochemical testing. The remaining two patients underwent aspiration biopsy, followed by microscopic observations and immunohistochemical testing.

## Results

### CT features

#### Tumor site and size

**Fig. 1:**
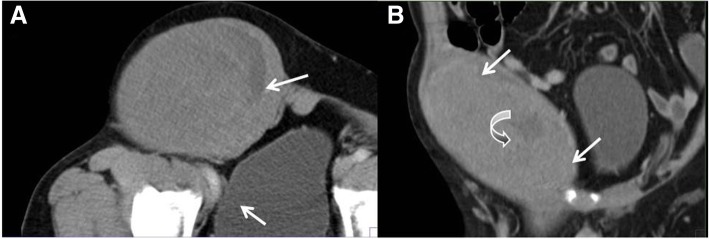
A 51-y-old male patient with an absent scrotum and testis on the right side. **a** Enhanced CT traverse scanning. **b** Coronal scanning. Solid masses with a complete capsule (arrow) were visible in the right pelvic cavity. These masses extended into the ipsilateral groin and led to bulging of the abdominal wall. The solid mass was slightly enhanced, and small low-density plaques (dots) were visible in the solid part (curved arrow). Surgery and pathology diagnosed an intra-abdominal undescended testis secondary seminoma

The tumors in all nine patients were unilateral and located in the right abdomen (*n* = 5), left abdomen (*n* = 2), or midline area (*n* = 2). In all cases, the tumor in the right abdomen was outward protruding to the abdominal wall and down growing to the groin (Fig. 1). In seven cases, the shape of the tumors was elliptical, and the long axis was consistent with the descending path of the testis. In two cases, the tumors were round shaped The maximum tumor volume was 145 mm × 97 mm × 229 mm (length × width ×width), and the minimum tumor volume was 60 mm × 55 mm × 65 mm.

#### Density

**Fig. 2:**
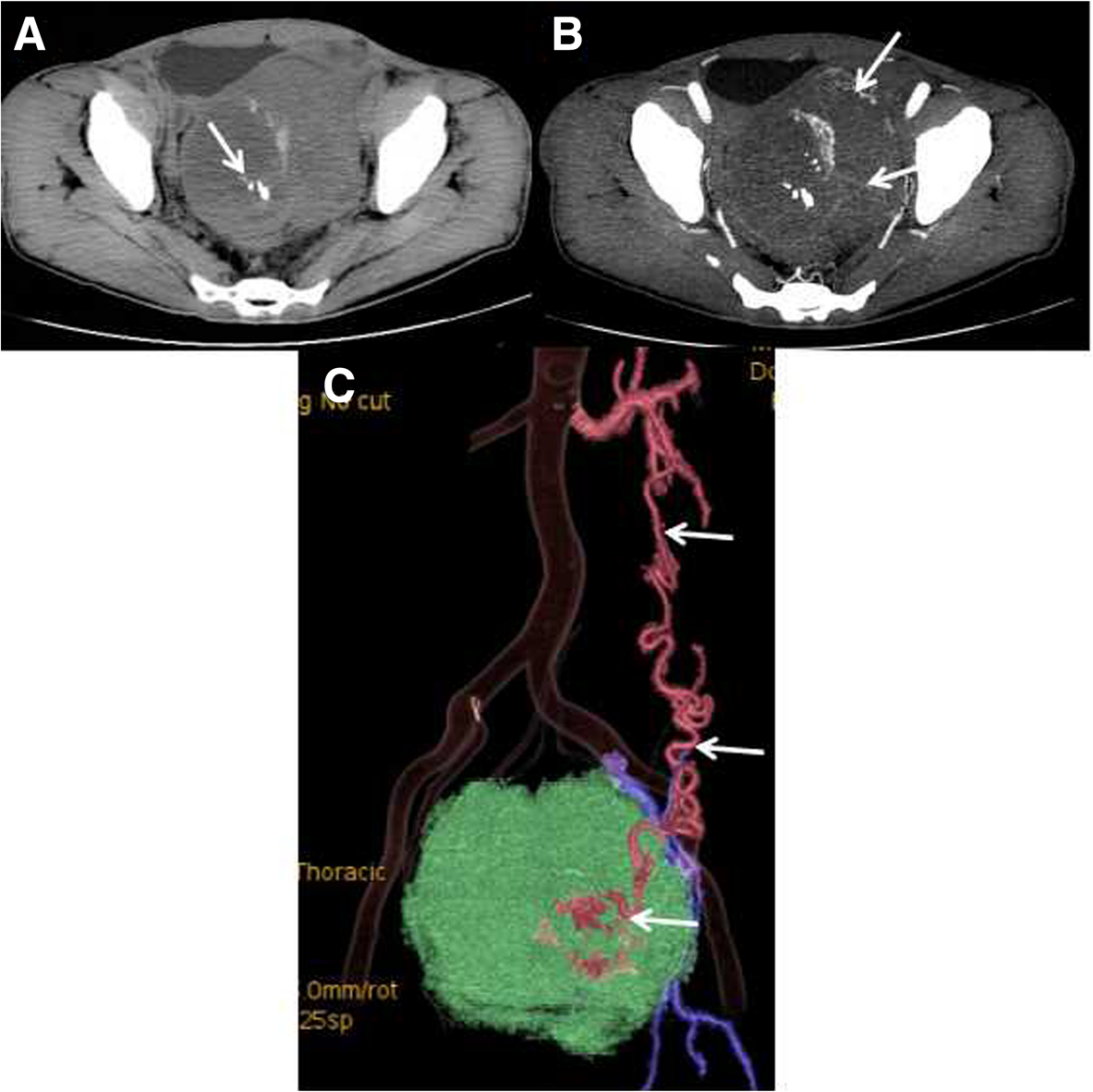
CT appearance of a pelvic undescended testis secondary seminoma. **a** A CT plain scan showed a huge and round soft tissue mass in the pelvis, with a clear margin and inhomogeneous density. Small dots or striped calcifications and irregular low-density areas were visible in the tumor. **b** In the arterial phase, vascular penetration was visible in the lesion (arrow). The solid part of the tumor was slightly enhanced. **c** SSD reconstruction showed that a thickened and tortuous left testicular artery was the supply artery of the tumor. The testicular arteries were clearly displayed, whereas the testicular veins were poorly displayed, which was consistent with the “testicular vascular pedicle sign”. “Testicular vascular pedicle sign” means testicular arteries were clearly displayed, whereas the testicular veins were poorly displayed, and the accessory blood vessels was relatively normal developed and displayed in contrast or enhanced CT

In the nine cases, most of the tumors were solid masses as shown by the CT scan. The CT value of the solid components was 38.4 ± 3.4HU (35–46 HU). The internal area was characterized by low-density lesions of various sizes. In two cases, dots or striped calcifications were detected inside the tumors (Fig. 2).

#### Enhancement

**Fig. 3:**
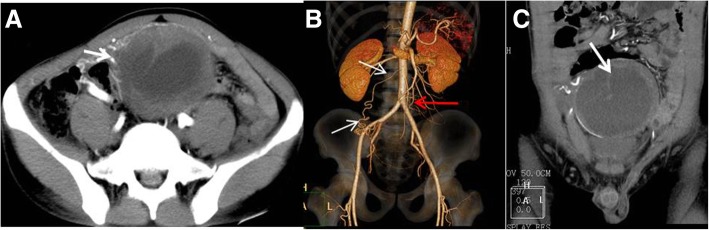
A 27-y-old male patient who had persistent abdominal pain for 2 wk. **a** A tumor located in the midline area of the lower abdomen. The tumor was oval, with a thickened tortuous artery visible at the right anterior lateral margin (arrow). **b** VR images showed that the supply arteries originated from the right anterior abdominal aortic wall 2 cm below the right renal ostia. The testicular arteries showed a dendritic distribution, with a branch-like shape (red arrow). The testicular arteries were clearly displayed, whereas the testicular veins were poorly displayed, which was consistent with the testicular vascular pedicle sign. **c** Coronal MPR showed a complete capsule (thin arrow) and a clear left spermatic cord (thick arrow). The right spermatic cord was absent

**Fig. 4:**
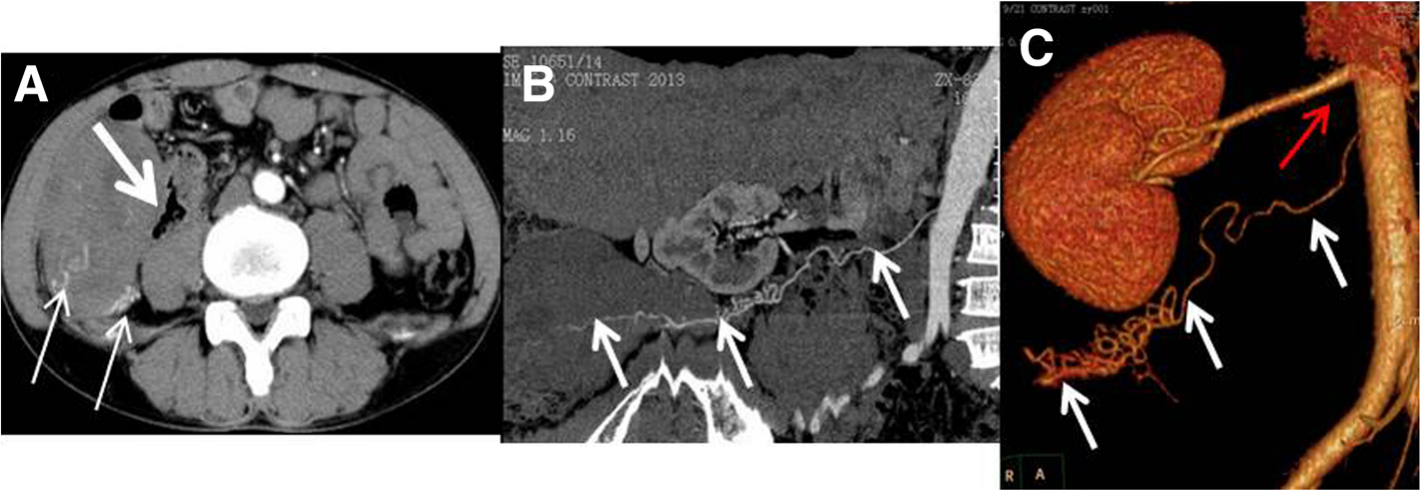
A 46-y-old male patient with right lumbago for 1 mo. **a** Tortuous supply arteries were visible in the outer posterior margin of the tumor and bended into the tumor (thin arrow). The boundary of the medial margin and jejunum was unclear (thick arrow). CPR (**b**) and VR (**c**) images showed that the supply arteries originated from the abdominal aorta 2 cm below the right renal artery, which was tortuous, with no branches (arrow). The testicular arteries showed a dendritic distribution, with a branch-like shape (red arrow). The testicular arteries were clearly displayed, whereas the testicular veins were poorly displayed, which was consistent with the testicular vascular pedicle sign

**Fig. 5:**
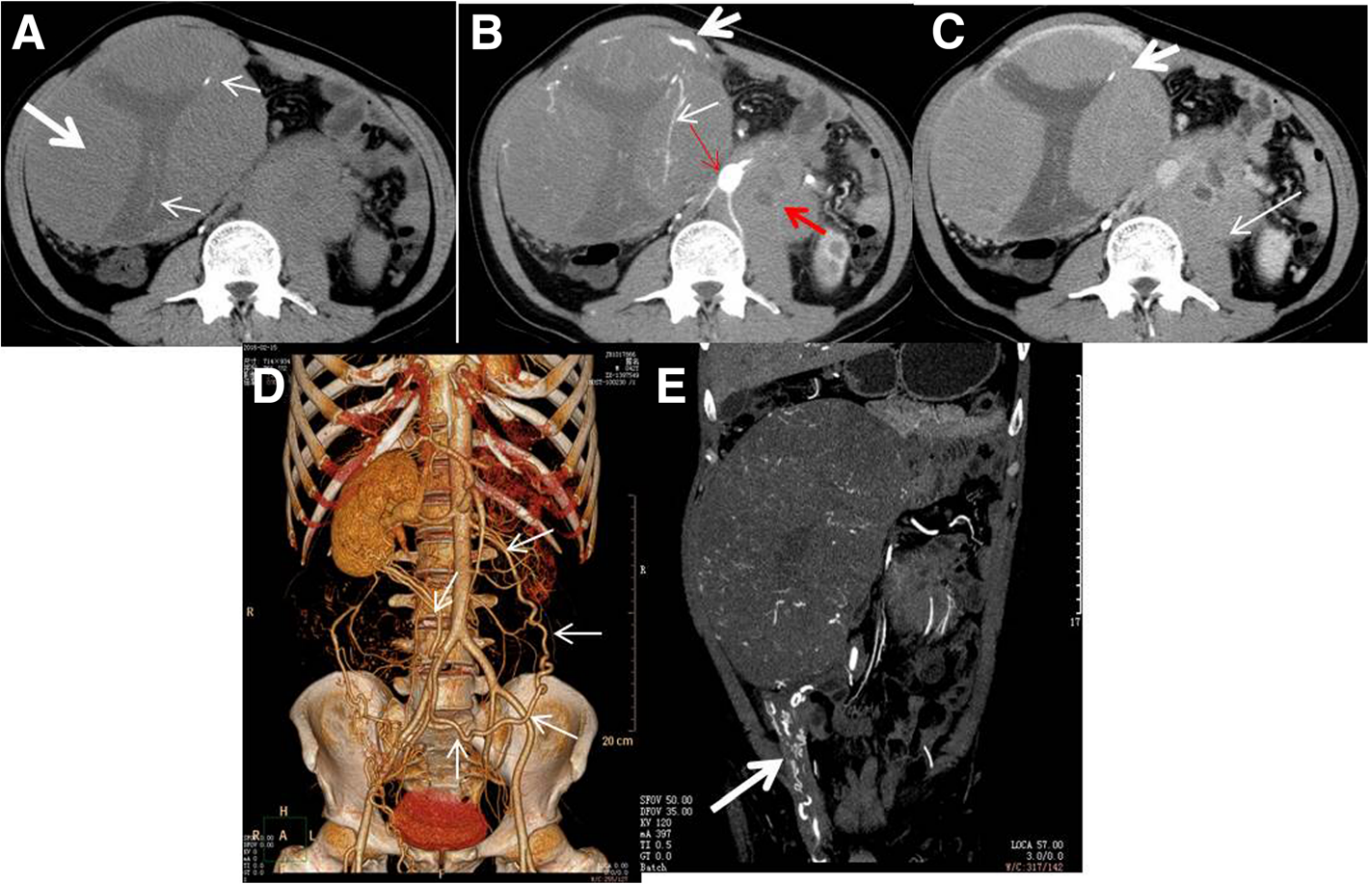
A 48-y-old male patient. **a** A CT plain scan showed that the tumor was mainly solid (thick arrow), with a CT value of 40 HU. A T-shaped low-density area was visible in the central area of the solid part of the tumor, and the CT value was 28 HU. Dots or striped calcifications were visible in the T-shaped low-density area (short thin arrow). **b** In the arterial phase, a thickened angiogram (arrow) was visible in the lateral margin of the tumor and distributed throughout the solid part of the tumor. The area containing the striped calcifications was slightly enhanced (thin arrow). Para-aortic lymph nodes were enlarged, and blood vessels were embedded. Vascular morphology was complete, and significant enhancement was found in the arterial phase (red thin arrow). Metastatic lymph nodes showed low density enhancement (red thick arrow), indicative of the floating ice sign. **c** The solid part of the tumor showed progressive enhancement, and no enhancement was seen in the low-density area. A tortuous artery was visible at the margin of the tumor (thick arrow). Enlarged lymph nodes showed progressive enhancement, and no enhancement was seen in necrotic areas (thin arrow). **d** The coronal reconstruction displayed a right varicocele (arrow), but the left varicocele was not visible. **e** VR images clearly displayed the origin and distribution of the blood vessels and the supply of the tumor (arrow)

**Fig. 6:**
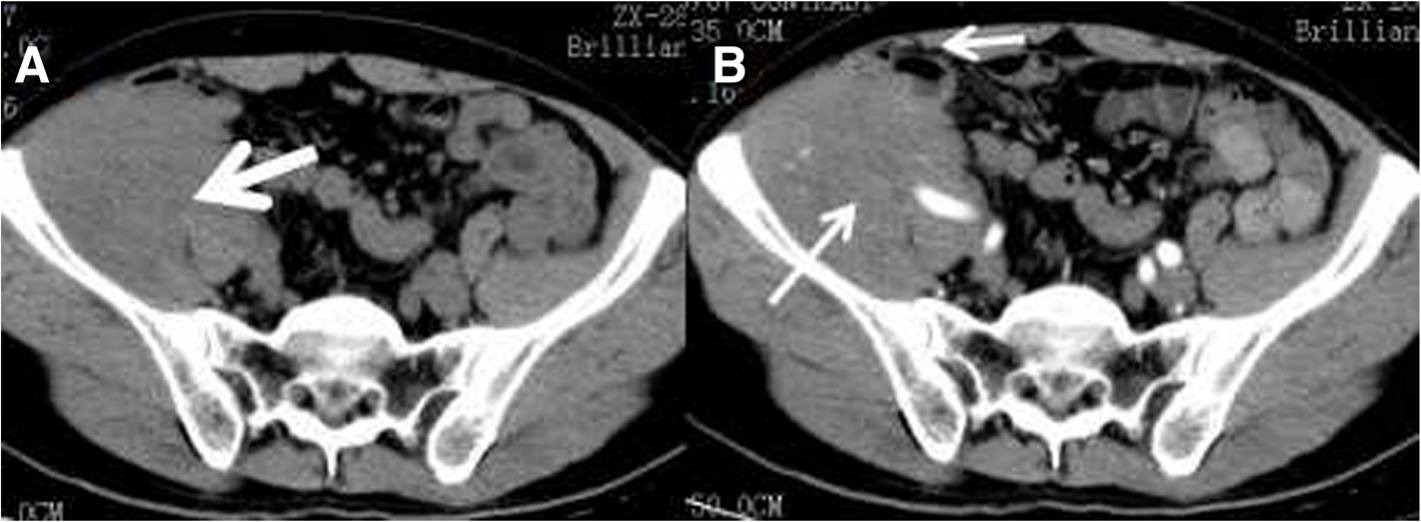
A right intra-abdominal undescended testis secondary seminoma. **a** A CT plain scan showed irregular soft tissue in the right iliac fossa (arrow). This tissue had a homogeneous density and a CT value of 38 HU. **b** In the enhanced arterial phase, the CT value of the lesion was 55 HU, and the blood vessels were embedded in the lesion. The boundary of the lesion and adjacent loop of bowel **(**short arrow)/iliacus (long arrow) were not clear, which was confirmed by biopsy at surgery

The solid parts of the tumors showed progressive enhancement. The CT values in the arterial phase, venous phase, and delayed phase were 46.3 ± 5.1 (40–55 HU), 57.3 ± 7.3HU (48–68 HU), and 65.1 ± 7.2 HU (56–77 HU), respectively, with an average maximum strengthening rate of 27.0 ± 7.2 HU. In all nine cases, on multiphase contrast-enhanced CT images, the tumors/lesions appeared as flaky, low-density areas, without obvious enhancement. In one case, a T-shaped low-density area was visible on contrast enhancement (Fig. 3). Enhanced angiogram distributed in arborescent type and a tortuous angiogram in the tumor margins was visible in the solid tumors in all cases (Figs. 3, 4 and 5). In the venous phase, increased testicular venous drainage was detected in three cases. A complete capsule was visible in the margins of the tumors in eight of the nine cases after enhancement. In the other case, imaging revealed an infiltrative tumor, with poorly defined boundaries, which was confirmed by biopsy at surgery (Fig. 6). After the biopsy, radiotherapy was performed on this patient. In this case, plain CT and enhancement CT showed soft tissue density in the right iliac fossa. Therefore, tumorous lesions were considered. Abdominal B-ultrasound revealed heterogeneous masses in the pelvic cavity, and contrast-enhanced ultrasound showed inhomogeneous enhancement. Thus, a tumor originating from the muscularis layer of the posterior pelvic wall was considered. A pathological examination showed a heterocyst in fibrous connective tissue. The findings pointed to a seminoma.

### CTA features

The CTA scan clearly showed the supply arteries in all nine cases. The testicular arteries in six cases originated from the right anterior abdominal aortic wall 2–3 cm below the right renal ostia. In three cases, the testicular arteries originated from the left anterior wall. The testicular arteries were tortuous, with no branching vessels (Fig. 2c). “Testicular vascular pedicle sign” [10] means testicular arteries were clearly displayed, whereas the testicular veins were poorly displayed, and the accessory blood vessels was relatively normal developed and displayed in contrast or enhanced CT (Figs. 2c, 3b, 4b, c). In eight patients, the testicular arteries originated from posterior of the tumor and showed a dendritic-like distribution (i.e., a branch-like shape) inside the tumor, or they were sparsely distributed in the tumor, without significant branches (Figs. 3b and 4c). In one patient with a large mass, the testicular arteries of the mass were thick, with a diameter of 2 mm, and they originated from the left anterior abdominal aortic wall 2 cm below the left renal artery. They traveled downward first and across the midline at the left external iliac artery before traveling upward and anterior to the tumor, finally culminating in the tumor (Fig. 5d). The location of the supply arteries was altered in this case because of the displacement and rotation of the huge mass.

#### Display of the testis and spermatic cord

In four patients whose tumors were located in the right abdomen, the ipsilateral testis and spermatic cord were absent in three cases, and abnormal development of the left testis and spermatic cord, with thickened tortuous spermatic vessels was present in one case (Fig. 5e). Combined with the CTA appearance, the tumor site and abnormal testis were not at the same side, which was related to the huge mass shifted to the right in the lower abdomen. The ipsilateral testis and spermatic cord were absent in three patients whose tumors were in the left abdomen. The right testis and spermatic cord were absent in two patients whose tumors were in the midline area (Fig. 3c).

#### Relationship between the tumor and surrounding structure

In all nine patients, the tumor oppressed the intestine in the abdominal cavity. In eight cases, there was a clear boundaryof tumor. In one case, there was an unclear boundary of tumor, with an adjacent loop of bowel and right iliac muscle (Fig. 6).

#### Lymph node metastasis

Posterior peritoneal lymph node metastasis was detected in two cases by progressive enhancement. The larger of these lymph nodes showed cystic necrosis (Fig. 5a-c). Among the lymph nodes embedded in the abdominal aorta and branches, the vascular morphology was complete on enhanced CT images, and significant enhancement was found in the arterial phase. The metastatic lymph nodes showed low density enhancement. Thus, the enhanced blood vessels were like ice floating on the sea, the so-called “floating ice sign” (Fig. 5b).

### Surgical pathology

In seven of the nine cases, the tumors had a complete capsule with a smooth surface, and they did not invade the surrounding structures. The section of the tumors appeared gray and contained necrotic areas. Gray fibrous scar tissue was visible in three tumors. In two cases, an aspiration biopsy was performed to enable a pathological examination.

**Fig. 7:**
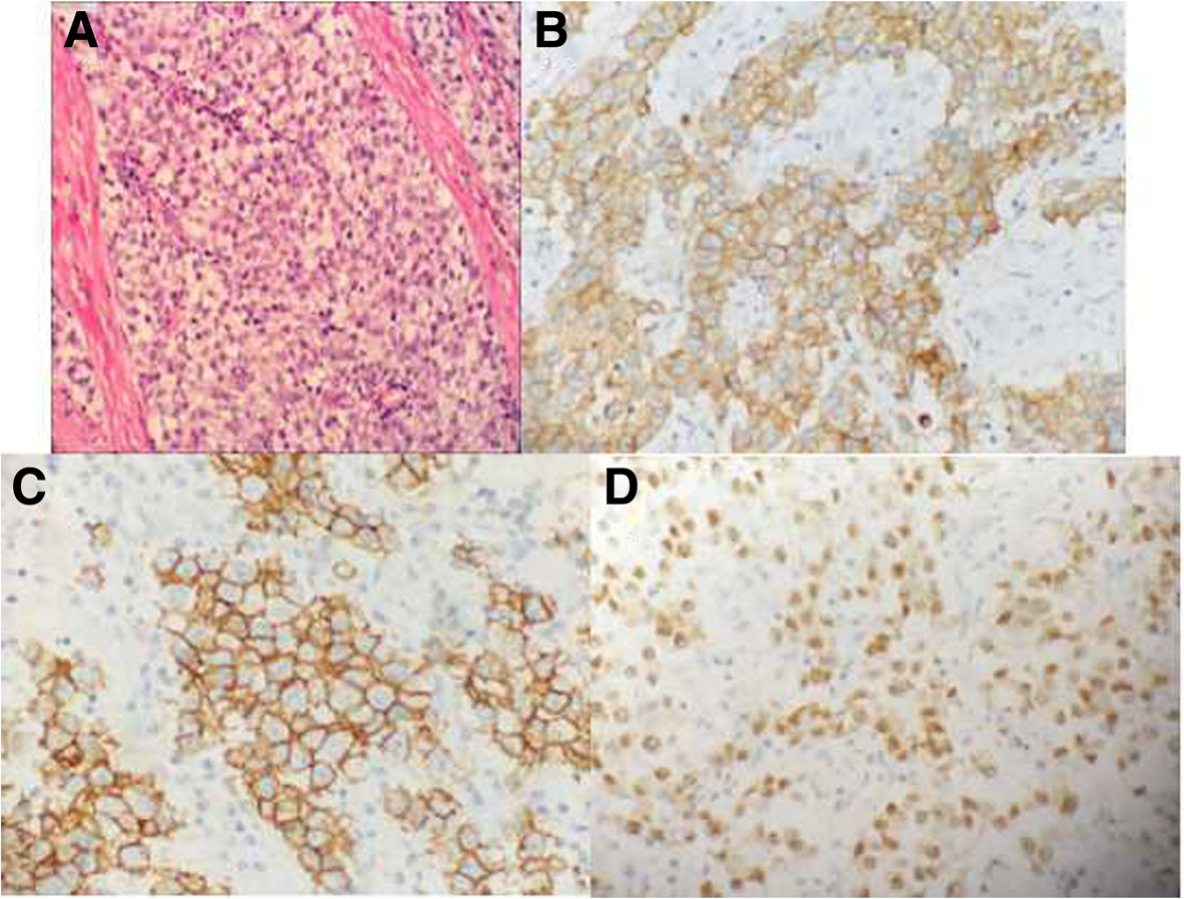
The same patient as shown in Fig. 4. **a** Under a microscope, single tumor cells had a nest-like arrangement, with similarly sized cells The cytoplasm was light colored. There was lymphocyte infiltration in the mesenchyme (HE× 10). **b** Immunohistochemistry indicated PLAP positive. **c** Immunohistochemistry indicated CD117 positive. **d** Immunohistochemistry indicated Oct3/4 positive

Under the microscope, single tumor cells showed a nest-like arrangement, and the size was almost the same. The color of its cytoplasm was light. The mesenchyme was characterized by lymphocyte infiltration (Fig. 7a). The results of immunohistochemistry were as follows: PLAP (++), CD117 (+++), Oct-3/4 (+++), D2–40 (+++), LCA (−), CD30 (−), CD20 (−), CD3 (−), CK20 (−), CK7 (−), ALK (−), EMA (−), Ki-67 (++) 60–70%, and S-100 (−). In all nine cases, the pathological diagnosis of the tumors was a seminoma (Figs. 7b-d).

## Discussion

A CT examination plays an important role in confirming an intraperitoneal undescended testis [11, 12]. Gubernaculum testis residue can indicate the location of an undescended testis [13, 14]. In previous studies, in cases of undescended testes secondary to tumors, the blood supply of the tumor originated from the testicular artery, and the tumor was drained by the testicular vein, as supported by angiography or enhanced CT, which showed normal development of accessory blood vessels in the undescended testis [2, 9, 10, 15]. In these cases, blockage of the testicle during the descending process led to an undescended testis. A unilateral undescended testis was common on the right side, which was mainly divided into intraperitoneal, inguinal, and scrotal type [16]. According to previous studies, tumors are common in cases of an intraperitoneal undescended testis, with seminomas accounting for 60% of such tumors [3, 9, 10]. Many factors can lead to malignant changes in an undescended testis, including the location, local temperature, blood circulation disorders, endocrine dysfunction, and gonadal agenesis. Early detection of the intraperitoneal type is difficult, even when the tumor is malignant. Furthermore, by the time the patient seeks medical attention, the intra-abdominal mass is often very large, leading to difficulties in making a qualitative diagnosis [17].

CT is useful in determining the properties of a seminoma [18]. Based on the literature and the data obtained in the present study, on CT imaging, an intra-abdominal undescended testis secondary seminoma can be diagnosed based on the following: 1) no ipsilateral testis and spermatic cord visible, 2) a testicular vascular pedicle, 3) growth orientation, 4) density and signal characteristics of the tumor, 5) a capsule, 6) an enhanced CT appearance, and 7) lymph node metastasis. In the present study, an ipsilateral testis and spermatic cord were visible in all the patients. Thus, the scrotum should be included in the CT scan. In the present study, on CTA, the testicular arteries were clearly displayed, whereas the testicular veins were poorly displayed (Figs. 2c, 3b, 4b and c). In previous studies, tumors in cases of an undescended testis. The accessory blood vessels showed a relatively normal development and were displayed in contrast or enhanced CT [10, 19, 20]. In the present study, the coronal/sagittal CT scan showed that the long axis of the tumor was consistent with the descending path of the testis. Thus, in cases of large tumors located in the abdominal cavity, displacement and torsion can be expected, together with possible changes in the orientation of the long axis of the tumor. In terms of the density and signal characteristics of seminomas, most of the tumors in this study were almost solid masses and were accompanied by cystic necrosis. Tumor necrosis was mainly present in the distal part of the tumor where the testicular artery penetrated the tumor. Calcifications were rare in the seminomas in the present study, with only two cases showing various shaped calcifications in the central part of the tumor.

Although a seminoma is a malignant tumor, the capsule is often complete, with expansive growth. In eight of the nine cases in the present study, the tumors had a complete capsule, and one of these cases had lymph node metastasis, without invasion of the surrounding structures. In one case, the tumor had an incomplete capsule and invaded the surrounding intestinal tubes. In this study, dendritic-vascular enhancement was present in all cases. Ueno et al. [3] proposed that dendritic-vascular enhancement was a characteristic manifestation of a seminoma and that the pathological basis was a fiber vascular stroma. In the present study, although there were abundant supply arteries, the degree of enhancement of the solid parts of the tumors was relatively low. This finding may be related to the blood-testis barrier in seminomas. According to a previous study, distant metastasis of seminomas in cases of an undescended testis was rare, but lymph node metastasis was often found in the abdominal aorta of the upper and lower plane of the renal hilus [21]. In this study, two patients had lymph node metastasis in the abdominal aorta in the abdominal aorta and the main arterial branches. The appearance was similar to that of a lymphoma and showed the floating ice sign. Lymph node metastases around a mass are uncommon, even in cases of secondary tumors, where the tumors occur in cryptorchidism or in the inguinal region. Tumors usually metastasize to the lymph nodes around the abdominal aorta in the plane of the renal hilus. According to a previous study, this may be explained by the direction of the lymphatic drainage and the abdominal aorta direction in the plane of the renal hilus [22].

In terms of the diagnostic value of CTA, as shown in this study, CTA can play an important role in the differential diagnosis of a seminoma secondary to an undescended testis. In arterial phase contrast enhancement, the images were reconstructed using a variety of post-processing methods, such as MPR, CPR, VR, and MIP, which clearly displayed the origin and distribution of the blood vessels. Thus, CTA is valuable for the diagnosis of the origin of the blood supply, preoperative positioning, and qualitative diagnosis of an intra-abdominal undescended testis secondary seminoma.

## Conclusion

As shown by the findings in the present cases, solid tumor with a complete capsule, the long axis of the tumor was consistent with the connection of the inferior ipsilateral renal and inner inguinal ring, tortuous vessels in the tumor margin on the enhanced scan, and dendrimer angiogram in the tumor, should raise a strong suspicion of an intra-abdominal undescended testis secondary to a seminoma. CTA can show the three-dimensional testicular vascular pedicle sign of a giant seminoma. It can shed light on the features of the tumor and the multidirectional relationship with the surrounding organs. A combination of CT and CTA can provide accurate and valuable information for the preoperative diagnosis and treatment.

## Abbreviations

CPR: Curved planar reformation
MIP: Maximum intensity projection
MPR: Multiplanar reformation
VR: Volume rendering
